# Long-term running in middle-aged men and intervertebral disc health, a cross-sectional pilot study

**DOI:** 10.1371/journal.pone.0229457

**Published:** 2020-02-21

**Authors:** Ulrike H. Mitchell, Jennifer A. Bowden, Robert E. Larson, Daniel L. Belavy, Patrick J. Owen

**Affiliations:** 1 Department of Exercise Sciences, Brigham Young University, Provo, Utah, United States of America; 2 Deakin University, Institute for Physical Activity and Nutrition, School of Exercise and Nutrition Sciences, Geelong, Victoria, Australia; Rush University Medical Center, UNITED STATES

## Abstract

**Purpose:**

To measure intervertebral disc (IVD) health parameters in middle-aged long-term runners compared to matched non-physically active controls.

**Methods:**

Seventeen males aged 44-62yr were included in the study: 9 runners with a running history of >10yr, averaging >50km/week, and eight matched non-physically active controls, the data from one participant had to be excluded. T2-relaxometry, diffusion weighted imaging, T1- and T2-weighted MR scanning, as well as T2 time mapping were performed. Morphological data relating to IVD were extrapolated.

**Results:**

Compared to controls on average, runners had 20% greater IVD height (p = 0.002) and seven percentage points greater IVD-vertebral body height ratio (p = 0.001). No significant differences were observed between groups for mean(SD) IVD hydration status, as indicated by similar T2-times (runners: 94.4(11.1)ms, controls: 88.6(23.6)ms), or apparent diffusion coefficients (runners: 249.0(175.2)mm^2^/s, controls: 202.3(149.5)mm^2^/s). Average Pfirrmann score for the L5-S1 IVD was 2.2(0.7) for runners and 3.3(1.0) for controls (p = 0.026), average scores for all lumbar levels (L2-S1) were 1.9(0.2) and 2.5(0.7), respectively (p = 0.036). Anterior annulus T2-time and overall average lumbar level Pfirrmann grades were strongly correlated (r = 0.787, p = 0.021 and r = -0.704, p = 0.034, respectively) with greater distances run per week. Average lumbar level Pfirrmann grades were also strongly correlated (r = -0.823, p = 0.006) to total years of running.

**Conclusion:**

Middle-aged long-term endurance runners exhibit less age-related decline in their lumbar IVDs. In addition, the measures of IVD morphology appeared to be better in those who had been running for a greater number of years, as well as in those who ran a greater distance per week.

## Introduction

Intervertebral disc (IVD) health is an important factor in overall spine health and the incidence of low back pain. It is strongly associated with nutrient flow within the disc [1]. In other tissues, nutrients are distributed to the cells through the cardiovascular system, however, IVD tissue is largely avascular in nature. While the outer annulus of the disc receives nutrients from the surrounding vasculature, the inner annulus and nucleus receive nutrients via bulk fluid flow within the disc tissue and diffusion through the vertebral endplates [2]. Multiple factors have been hypothesized to affect nutrient flow and thus disc health. For example, physical activity level and other lifestyle factors, such as high amounts of sedentary time, both in occupational and recreational time, influence disc nutrient flow [2–4]. Moreover, loading and resulting compression and deformation of the disc leads to expulsion of bulk fluid, while distraction encourages uptake of fluid and nutrition [5, 6]. As the IVD ages, its morphology changes and it loses its ability to attract water, rendering it more susceptible to injury [7]. After the age of 30 there is an increased rate of normal age-related IVD degeneration and the peak for these degenerative changes occurs around the age of 50 [8].

IVD health can be assessed using magnetic resonance imaging (MRI) [9]. T1 and T2 imaging techniques are useful for observing morphological changes to the IVD. In addition, the images can be used to grade and categorize the level of disc degeneration (Pfirrmann grading system [9]). Marinelli et al. [10] showed that T2 relaxation time, an intrinsic property of tissue that can be measured with MRI, significantly and strongly correlated with water content in the IVD. Belavý et al. [11] used T2 relaxation times to demonstrate that chronic runners aged 25-35yr exhibited better IVD tissue quality (i.e. hydration) compared to non-physically active controls. Diffusion-weighted imaging, a more advanced MRI application, has allowed clinicians and researchers to evaluate real time water movement within the IVD [12]. It gives a snapshot of the overall fluid movement within the disc and provides an earlier indication of changes in disc tissue than T2 relaxation time. Diffusion-weighted imaging can also be used to calculate the apparent diffusion coefficient (ADC), a value that reflects the average diffusion rate of water molecules [13].

Running is an activity that is of particular interest in examining disc health, as there are substantial compression and rotational forces placed on the lumbar disc with running, which likely impact disc health. Depending on the magnitude, frequency and duration of the forces, they can have an anabolic effect on the IVD [14]; however prolonged dynamic loading has been shown to bring about signs of degeneration [15]. Dimiatriadis et al. [16] showed that runners undergo an average disc height decrease of almost 1mm in each of the lumbar IVD after 1h of running. This could be interpreted as being detrimental to the structure of the disc, or as a benefit to the metabolic processes. Therefore, two competing theories concerning loading of the spine and its consequences to IVD health were considered in this study: 1) cyclic loading is beneficial for IVD tissue and leads to hypertrophic changes that make the disc stronger [11], and 2) mechanical overload produces localized trauma and tissue damage, which outpaces the ability of a disc to repair itself and leads to accelerated degradation [14]. Both theories are based on robust previous research, but the implications are conflicting.

The aim of this pilot project was to assess if IVD parameters obtained from MRI scans of middle-aged long-term runners and matched non-physically active controls are different and as such invite further studies. The particular IVD parameters were: T2-relaxation time, ADC, disc height (relative to vertebral body height, an internal control to body size [2]), and disc degeneration (Pfirrmann grade).

## Methods

The study was approved by the institutional internal review board of Brigham Young University and participants provided their written informed consent prior to participation. A cross-sectional study was conducted from March to September 2018. We hypothesized that we will find different IVD parameters obtained from MRI scans for each of our two groups.

### Description of participants

This study included men aged 44-62yr. By this age, IVD tissue alterations due to normal age-related IVD degeneration should be visible and discernible on MRI.

#### Inclusion criteria

Male volunteers aged 44-62yr; the members in the running group had to have a history of running for >10yr, and had to have run on average >50km/wk. Members in the comparison group (control) had to have performed less than 150min/wk moderate physical activity, walk less than 15min to or from their place of work and not have performed regular sport or exercise training in the past 10yr.

#### Exclusion criteria

Participants were excluded if they had current spinal pain greater than 4 on the digital pain scale, have a history of spinal surgery, history of traumatic injury to the spine, known scoliosis or kyphosis for which prior medical consultation was sought, and being a current or prior smoker. Women were excluded as they have a greater predisposition to back-related problems compared to men because of sex-specific events. These include accelerated post-menopausal disc degeneration [17] and increased propensity to vertebral fracture [18] in post-menopausal years. Participants were also excluded based on contraindications for MRI, such as known claustrophobia, metal object in the body, pacemaker or implantable cardioverter defibrillator.

#### Recruitment

Participants were recruited by word of mouth. After running group participants were enrolled, we recruited an age-, sex- and height-matched control group. The controls were matched in order of priority to height (within 5cm), age (within 3yr), weight (within 3kg) and BMI (within 1kg/m^2^). Each subject had to match in at least two of the categories. All data were collected between noon and 3pm to account for diurnal changes in disc height and hydration status.

### Measures

#### Magnetic resonance imaging (MRI), image processing and analysis

We used a 3-Tesla Siemens MAGNETOM Tim Trio (Siemens Healthineers, Erlangen Germany) scanner with a 4-channel flexible coil. To quantify bone and IVD morphology, a sagittal T1-weighted scan was taken. For determination of IVD degeneration level (Pfirrmann grade) we took a sagittal T2-weighted turbo-spin echo sequence. A T2 mapping multi-echo spin echo (MESE) sequence was used to quantify T2 time, and a transverse two-dimensional diffusion weighted image was used to evaluate ADC for the L5-S1 IVD. Specific parameters for all MRI imaging are included in Table 1.

**Table 1:** MRI parameters.

**1. Sagittal view two-dimensional T1 weighted turbo-spin echo sequence**	
Field of View (FOV) readout-phase	256mm x 256mm
Matrix size kx-ky-slice	512 x 512 (phase encode 100% oversample) x 1
Voxel size x-y-slice thickness	0.5mm x 0.5mm x 5mm
TR/TE/Flip angle	700/11 msec/flip angle is 150 degree
Other parameters: Fat suppression, Turbo factor = 6, echo trains per slice = 170, readout bandwidth = 250 Hz/pixel	
**2. Sagittal view two-dimensional T2 weighted turbo-spin echo sequence**	
Field of View (FOV) readout-phase	280mm x 280mm
Matrix size kx-ky-slice	384 x 288 (phase encode 100% oversample) x 13
Voxel size x-y-slice thickness	1.3mm x 1.3mm x 4mm
TR/TE/Flip angle	3500/99msec/flip angle is 160 degree
Other parameters: Turbo factor = 32, slice gap is 1.3mm, readout bandwidth = 260 Hz/pixel, Average = 2, flow compensation is applied in readout direction.	
**3. T2 mapping multi-echo spin echo (MESE) sequence**	
Field of View (FOV) readout-phase	256mm x 256mm
Matrix size kx-ky-slice	384 x 288 (phase encode 100% oversample) x 20
Voxel size x-y-slice thickness	1.3mm x 1.3mm x 4mm
TR/TE/Flip angle	2700/11, 22, 33, 44, 55ms/flip angle is 180 degree
**4. Two-dimensional DWI sequence**	
Field of View (FOV) readout-phase	200mm x 200mm (phase with 100% oversample)
matrix size kx-ky-slice	128 x 128 (with 6/8 phase partial Fourier) x 1
Voxel size x-y-slice thickness	2mm x 2mm x 5mm
TR/TE/echo spacing	3000/113/0.73msec

Other parameters

Average = 4, readout bandwidth = 1502Hz/pixel

Diffusion directions = 64, b value = 1000s/mm2

After obtaining images in DICOM format, they were loaded and viewed on OsiriX (Pixmeo, Geneva, Switzerland). A mid-sagittal plane view was used and the IVDs of interest were manually segmented from the 2D T1 structural scan. T2 relaxation times and ADC values were determined for each IVD of interest.

Two investigators (UHM and REL) determined Pfirrmann grades of each lumbar IVD on sagittal T2-weighted images independently and in a blinded fashion. When the two scores did not coincide, the assessors discussed the cases and came to an agreement.

**Fig 1:**
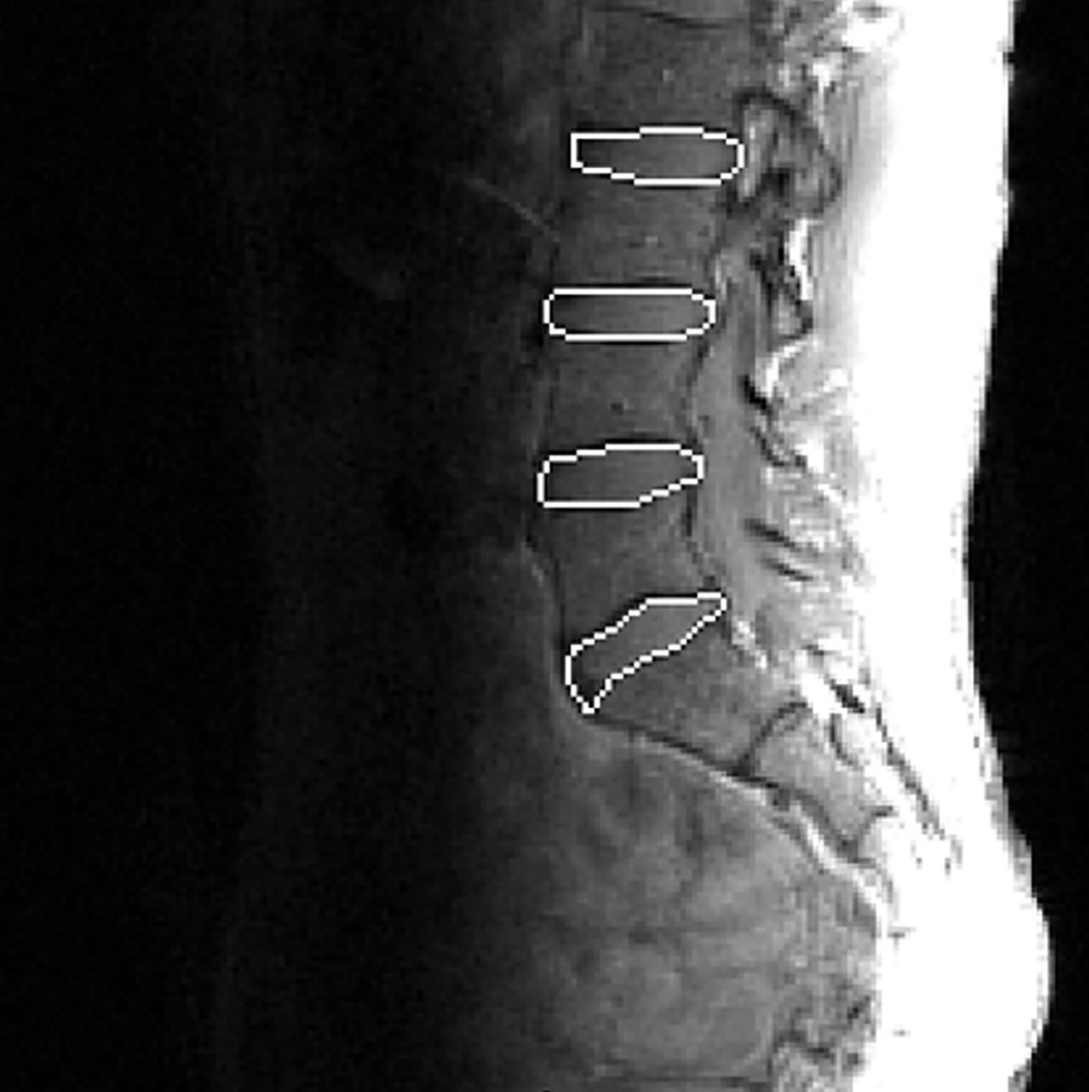
Example diagram showing IVD ROIs on one MR image.

ImageJ 1.38x (http://rsb.info.nih.gov/ij/) was used to perform T2 time analysis. All available IVD from L2-S1 were measured. After segmenting the IVD, a custom written ImageJ plugin (“ROI Analyzer”; https://github.com/tjrantal/RoiAnalyzer) was used to measure area, height, width and signal intensity of the IVD in its entirety as well as in five subregions from the anterior to posterior aspect of the disc (Fig 1). T2-time was calculated via linear fit to the natural logarithm of the image intensity in each of the available MR echoes.

### Statistical analyses

All analyses were conducted using Stata statistical software version 15 (College Station TX, USA). Measures were compared between groups by one-way analysis of variance. In an exploratory analysis, the strength and direction of associations between running activity (runners only) and outcomes of interest were assessed with Pearson correlation coefficient. Analyses considered outcomes averaged across all lumbar vertebral levels (L2-S1) or each individual level, except ADC which used L5-S1 only [19]. An alpha-level of 0.05 was adopted for all statistical tests.

## Results

Seventeen participants were included in analyses (runners: n = 9; control: n = 8). One participant in the control group complained of claustrophobia and we ended the scan pre-maturely. We had to consequently exclude his data. Participant demographics, including level of disability and health status, as measured by the Oswestry Disability questionnaire [20], health-related quality of life domains (36-Item Short Form Survey Australian version; SF36V2 [21]), and running activity are shown in Table 2. On average, controls had 9.7% greater body mass index (BMI) than runners, despite no significant difference in height or weight. The range of running activity parameters were: 10-39yr, 56-129km/wk, 4-6d/wk and 3.9–4.8min/km. No differences were observed in subjective measures of physical function or health-related quality of life domains.

**Table 2:** Participants demographics and running activity in runners (n = 9) and controls (n = 8).

	**Runners (n = 9)**	**Control (n = 8)**	**P-value**
Age, years	48 (4)	50 (4)	0.437
Height, cm	179.2 (5.1)	175.4 (5.1)	0.151
Weight, kg	68.9 (4.9)	72.0 (7.1)	0.308
Body mass index, kg/m^2^	21.6 (1.1)	23.7 (2.3)	**0.032**
Oswestry Disability Index, %	3.75 (6.88)	6.29 (6.47)	0.477
36-Item Short Form Survey, points (0–100)			
Physical functioning	96.25 (10.61)	95.00 (5.00)	0.781
Role limitation: Physical	93.75 (17.68)	98.29 (4.54)	0.522
Role limitation: Emotional	90.63 (17.52)	98.86 (3.02)	0.244
Energy/fatigue	73.50 (21.87)	69.71 (9.03)	0.677
Emotional well-being	85.00 (15.12)	85.71 (9.76)	0.917
Social functioning	90.63 (26.52)	94.71 (9.78)	0.707
Pain	86.63 (18.43)	87.71 (11.51)	0.895
General health	85.63 (18.02)	77.14 (11.85)	0.309
Running			
Years	23 (13)	-	-
Kilometres per week	82.6 (27.9)	-	-
Days per week	6 (1)	-	-
Minutes per kilometre	4.3 (0.4)	-	-

Data are mean (standard deviation).

**Fig 2:**
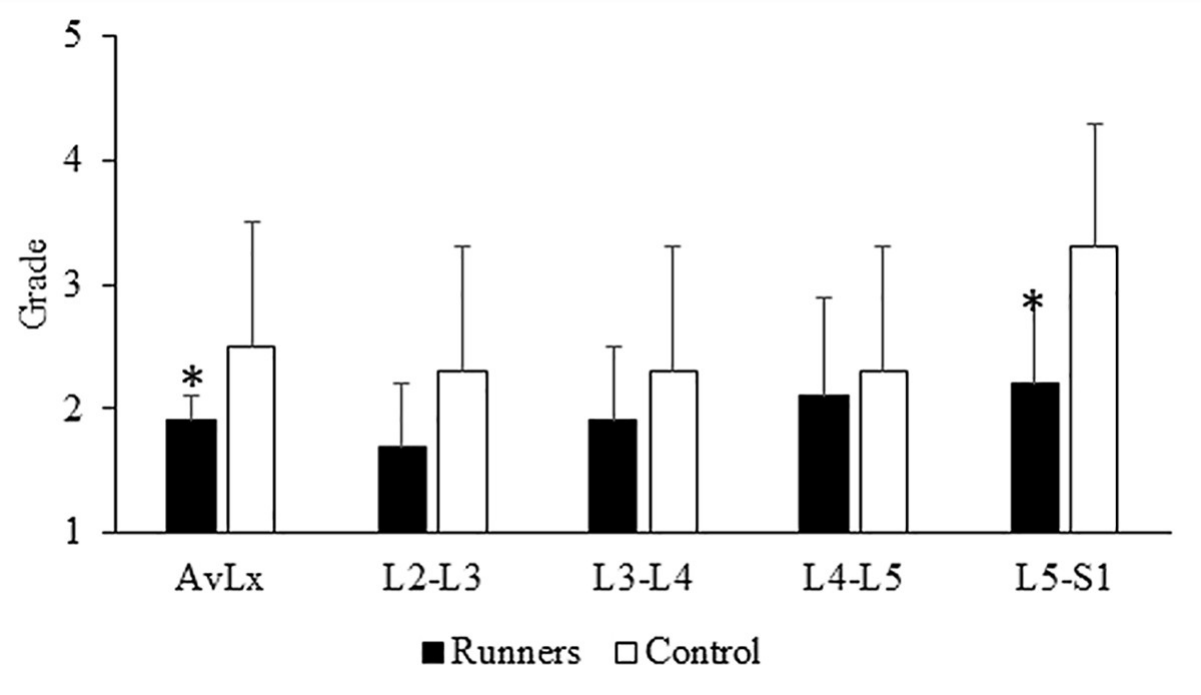
Average and individual lumbar IVD Pfirrmann grades. *P<0.05 when compared to control Average Pfirrmann grades for the whole lumbar spine and the L5/S1 segment were approximately half a grade significantly lower in runners compared to controls.

Average lumbar level morphology of the vertebral body and IVD and outcomes of IVD health are displayed in Table 3. Compared to controls runners had significantly lower (4.4%) vertebral body height, greater (20%) IVD height and greater (seven percentage points) IVD-vertebral body height ratio. No differences were observed between groups for T2-time or ADC. Average Pfirrmann score was approximately half a grade significantly lower in runners compared to controls (Fig 2). Notably, additional analyses using linear mixed models with random effects for within participant variance across all lumbar levels did not alter these results (Table 3).

**Table 3:** Vertebral body and intervertebral disc (IVD) morphology and IVD health outcomes in runners (n = 9) and controls (n = 8).

	**Primary analysis**			**Secondary analysis**		
	Runners (n = 9)	Control (n = 8)	P-value	Runners (n = 9)	Control (n = 8)	P-value
Vertebral body height, mm	26.8 (1.0)	28.0 (1.0)	**0.043**	26.8 (1.4)	28.0 (1.5)	**0.016**
IVD height, mm	8.3 (0.7)	6.8 (0.7)	**0.002**	8.3 (1.2)	6.8 (1.0)	**<0.001**
IVD-vertebral body height ratio	0.31 (0.04)	0.24 (0.02)	**0.001**	0.31 (0.05)	0.24 (0.03)	**<0.001**
IVD T2-time, ms						
Total	91.3 (11.2)	86.8 (23.9)	0.649	91.3 (14.9)	86.8 (24.5)	0.610
Anterior annulus	73.2 (6.1)	75.7 (19.2)	0.731	73.2 (7.3)	75.7 (18.2)	0.702
Anterior nucleus	89.1 (12.6)	84.3 (22.2)	0.625	89.1 (16.6)	84.3 (23.9)	0.584
Centre nucleus	101.7 (13.3)	92.5 (24.9)	0.396	101.7 (20.5)	92.5 (27.7)	0.336
Posterior nucleus	96.8 (14.6)	89.1 (25.1)	0.491	96.8 (19.5)	89.1 (27.1)	0.439
Posterior annulus	78.4 (5.8)	78.1 (19.3)	0.970	78.4 (7.2)	78.1 (18.7)	0.966
Apparent diffusion coefficient, mm^2^/s	249.0 (175.2)	202.3 (149.5)	0.566	-	-	-
Pfirrmann, grade (1–5)	1.9 (0.2)	2.5 (0.7)	**0.036**	1.9 (0.6)	2.5 (0.9)	**0.015**

All data are mean (standard deviation). Primary analyses were independent t-tests using the average of lumbar levels (L2-S1). Secondary analyses were linear mixed models with random effects for within participant variance across all lumbar levels (L2-S1). Apparent diffusion coefficient was measured at L5-S1 only.

Lumbar level-specific vertebral body and IVD morphology are shown in Table 4. Runners had 20% and 30% significantly greater IVD height, for L2-L3 and L3-L4, respectively, compared to controls. Moreover, IVD-vertebral body height ratio was significantly greater in runners than controls for L2-L3 (36%), L3-L4 (25%) and L5-S1 (24%). Pfirrmann score was also one grade significantly lower for level L5-S1 when comparing runners to controls.

**Table 4:** Vertebral body and intervertebral disc (IVD) morphology and IVD health outcomes in runners (n = 9) and controls (n = 8) by lumbar level.

	**Runners (n = 9)**	**Control (n = 8)**	**P-value**
Vertebral body height, mm			
L2-L3	26.8 (1.0)	28.0 (1.0)	0.284
L3-L4	26.6 (1.3)	27.4 (1.6)	0.074
L4-L5	26.7 (1.6)	28.2 (1.3)	0.319
L5-S1	27.5 (1.1)	28.3 (1.9)	0.072
IVD height, mm			
L2-L3	8.3 (0.7)	6.8 (0.7)	**<0.001**
L3-L4	8.7 (1.0)	6.4 (0.8)	**0.009**
L4-L5	8.6 (1.0)	7.0 (0.9)	0.122
L5-S1	8.5 (1.4)	7.4 (1.0)	0.108
IVD-vertebral body height ratio			
L2-L3	0.33 (0.04)	0.23 (0.02)	**<0.001**
L3-L4	0.32 (0.05)	0.25 (0.03)	**0.009**
L4-L5	0.31 (0.06)	0.26 (0.04)	0.085
L5-S1	0.28 (0.05)	0.22 (0.04)	**0.043**
IVD T2-time, ms			
Total			
L2-L3	101.2 (13.6)	93.1 (26.6)	0.479
L3-L4	95.1 (13.9)	89.6 (26.8)	0.629
L4-L5	83.9 (12.2)	85.7 (32.7)	0.888
L5-S1	85.1 (14.8)	78.8 (14.9)	0.471
Anterior annulus			
L2-L3	74.8 (8)	76.7 (18.9)	0.807
L3-L4	73.0 (5.8)	75.6 (17.1)	0.703
L4-L5	73.0 (6.8)	74.5 (22.1)	0.855
L5-S1	71.9 (9.3)	76.1 (21)	0.627
Anterior nucleus			
L2-L3	97.0 (13.3)	90.0 (26.4)	0.536
L3-L4	94.0 (15.9)	87.3 (21.6)	0.529
L4-L5	82.7 (15.1)	85.6 (33.5)	0.836
L5-S1	82.5 (19.2)	74.2 (15.4)	0.429
Centre nucleus			
L2-L3	115.1 (19.3)	101.6 (28.3)	0.324
L3-L4	109.1 (17.4)	96.9 (29.3)	0.361
L4-L5	91.3 (15.0)	91.9 (37.0)	0.970
L5-S1	91.4 (21.3)	79.6 (16.4)	0.313
Posterior nucleus			
L2-L3	112.6 (18.3)	97.2 (29.2)	0.261
L3-L4	99.9 (18.6)	93.0 (29.6)	0.612
L4-L5	86.1 (16.0)	87.9 (36.8)	0.906
L5-S1	88.7 (15.5)	78.3 (12.6)	0.234
Posterior annulus			
L2-L3	79.8 (8.4)	78.6 (20.2)	0.881
L3-L4	77.8 (7.9)	77.1 (23.6)	0.939
L4-L5	75.3 (5.9)	73.5 (19.7)	0.817
L5-S1	80.8 (6.5)	83.3 (15.7)	0.690
Pfirrmann, grade (1–5)			
L2-L3	1.7 (0.5)	2.3 (0.7)	0.066
L3-L4	1.9 (0.6)	2.3 (0.5)	0.190
L4-L5	2.1 (0.8)	2.3 (1.0)	0.758
L5-S1	2.2 (0.7)	3.3 (1.0)	**0.026**

Data are mean (standard deviation) by lumbar level.

Associations between running activity and outcomes across all lumbar levels are displayed in Table 5. Greater total years of running (P = 0.006), kilometres ran per week (P = 0.034) and minutes per kilometre (P = 0.044) were associated with lower Pfirrmann grades. Greater kilometres ran per week was also associated with longer IVD anterior annulus T2-times (P = 0.021).

**Table 5:** Correlations between variables examined in runners (n = 9).

	Correlation for variable (running activity):			
Variable	Years	Kilometres per week	Days per week	Minutes per kilometre
Vertebral body height	-0.077	-0.694	0.162	-0.494
IVD height	0.219	0.397	0.112	-0.099
IVD-vertebral body height ratio	0.206	0.539	0.034	0.169
IVD T2-time				
Total	0.361	0.568	0.400	-0.190
Anterior annulus	0.319	0.787*	0.464	0.145
Anterior nucleus	0.398	0.552	0.365	-0.671
Centre nucleus	0.319	0.517	0.386	-0.320
Posterior nucleus	0.395	0.522	0.411	-0.342
Posterior annulus	0.190	0.573	0.217	0.482
Apparent diffusion coefficient	0.439	-0.168	0.357	-0.251
Pfirrmann	**-0.823†**	-0.704*	-0.474	-0.768*

Data are Pearson’s correlation coefficient and average of lumbar levels.

* P<0.05

† P<0.01

‡ P<0.001.

## Discussion

The results of this study demonstrate that MRI scans of middle-aged long term endurance runners are different to scans from matched non-runners. The main finding was that long-term running has a positive effect on IVD health. Specifically, the current study showed that runners had lower average lumbar spine Pfirrmann grade and greater IVD-to-vertebral body height ratio. Moreover, these measures of IVD health appeared to be better in those who had been running for a greater number of years, as well as those who ran a greater distance per week.

The IVD of runners demonstrated less age-related degeneration, as measured by Pfirrmann grades [9]. The 5-point grading system is based on T2-weighted MR images and the structure of the IVD is taken into consideration. An IVD with a grade 1 represents a healthy, non-degenerated disc, while a grade 5 represents a severely degenerated IVD. In our study, runners exhibited healthier IVD, as demonstrated by lower Pfirrmann grades, compared to the non-physically active control participants across all lumbar levels, as well as specifically at the L5-S1 IVD. This finding is of particular interest, because the lower lumbar levels (i.e. L4-L5 and L5-S1) have been found to be the most common sites for disc degeneration[22] and disc herniation[23]. In contrast to our findings, Belavý et al. [11] found that there was no difference in Pfirrmann grades between participants who were not regularly involved in sport compared to runners. Their population was an average of 20yr younger, and morphological changes due to age and activity may have not yet manifested themselves (at least not on MR images). Our findings suggest that long-term running may delay IVD degeneration or possibly result in beneficial modulation of this tissue.

Runners in the current study had a greater IVD-to-vertebral body height ratio compared to their non-physically active counterparts. The difference in ratios was a function of both a smaller vertebral body height and a larger IVD height in the runners. Belavý et al. [11] also found that long distance runners, but not the joggers, nor the participants in the non-sporting group, exhibited a greater IVD-to-vertebral body height ratio and referred to this as ‘IVD hypertrophy’. This finding is interesting because it is commonly accepted that lumbar IVDs decrease in height with advancing age [24] as a result of morphology changes and the reduced ability to bind water [7]. On the other hand, endurance running may just be delaying degeneration and not actually causing disc growth. Somewhat related to this are findings by Videman et al.[25] who reported that the heavier twin in weight discordant monozygotic twins exhibited slightly larger IVDs compared to their lighter twin. This was explained by the greater habitual loading the heavier twin experienced, a concept similarly proposed to explain why individuals with greater fat mass often have greater absolute muscle mass. In our study the vertebral body height was smaller in runners compared to their non-sporting counterparts. Therefore, long-term running (i.e. habitual dynamic axial loading of the spine) may have elicited a bone response with possible physiologic changes in bone architecture. We do not have data on bone architecture, nor density, of any of the vertebrae, thus our suggestions that the decrease in vertebral height was coupled with increased bone density remains speculative. Nonetheless, these morphological observations in the current study support that running is associated with IVD hypertrophy and thus better IVD health.

Both greater total years of running and total distance ran per week were associated with lower Pfirrmann grades, which indicates that greater amounts of running may delay IVD degeneration. Similarly, there was a positive association between running distance and T2-times, indicating greater IVD hydration, albeit this was only observed in the annulus. The annulus contains substantially fewer water binding proteoglycans compared to the nucleus, which makes this finding remarkable.

Surprisingly, parameters in the current study indicating hydration of the disc (i.e. T2-time and ADC) did not differ between groups, despite differences in Pfirrmann grade. Previously, Belavý et al. [11] found significantly longer T2-times in runners compared to non-athletic individuals. The discrepancy between our findings may be explained by the age difference of our participants. Our study used participants with an average age of 50yr (20yr older than comparator), when disc hydration had been found to be greatly reduced [26]. We intentionally recruited middle-aged participants, to assess if habitual cyclic loading of the disc was able to delay the desiccation process associated with ageing [7]. However, the data from our current study do not support the notion that running delays this process. Nevertheless, our data indicate longer average T2 times in all areas of the IVD and at all levels in the runners compared to the controls, except for the anterior annulus. This difference is not statistically significant, most likely because the data are likely underpowered and have high variability.

The results of this cross-sectional study confirmed previous findings [11] that there is an association between long-term long-distance running and better IVD health. They also support earlier speculations that the IVD is able to undergo anabolic adaptations within a human life span [11]. The question of an optimal loading pattern, however, remains elusive. The following information should be used to elucidate this matter in further studies: The relationship between habitual physical activity and risk of IVD degeneration has been described as U-shaped [27], indicating that either too much or insufficient loading of the spine is detrimental to IVD health. In addition, it has been suggested that there might be a ‘physiological range’, or ‘zone’ of dynamic axial compressive loads that lead to an anabolic response within the IVD [27]. This also implies that there might exist a ‘therapeutic window’ of optimal benefits one can receive from running. Establishing these parameters has potential public health implications as IVD degeneration is a leading cause of mechanical low back pain. Therefore, interventional exercise training studies examining the efficacy of running for IVD health are warranted.

Our study was strengthened by the blinded nature of MRI analyses, as well as the matched-nature of controls. Cross-sectional studies, however, have well known limitations. Despite observations that running was associated with better IVD health, we cannot draw a cause and effect conclusion (e.g. running is the cause for healthy IVDs). Similarly, we cannot dismiss the possibility that there is reverse causality (e.g. healthier discs allow people to continue running, while degenerated discs force people to stop). Another limitation was the variance in running activity (e.g. range of years: 10-39yr). Lastly, while we explained why only men were included in this study, these findings may not apply to women.

## Conclusion

Middle-aged long-term endurance runners exhibit less age-related decline in their lumbar IVDs compared to matched non-runners. In addition, the measures of IVD morphology appeared to be better in those who had been running for a greater number of years, as well as in those who ran a greater distance per week. Our findings support those of the only other study that investigated the effect of long term running on the IVD [11] and broaden the implications to an older and more active population.

## Supporting information

S1 Table Supporting data spreadsheet. (XLSX)
